# Novel High-Entropy Aluminide-Silicide Alloy

**DOI:** 10.3390/ma14133541

**Published:** 2021-06-25

**Authors:** Pavel Novák, Kateřina Nová

**Affiliations:** 1 Department of Metals and Corrosion Engineering, University of Chemistry and Technology, Prague, Technická 5, 166 28 Prague 6, Czech Republic; knova@kmm.zcu.cz; 2 Department of Materials and Engineering Metallurgy, University of West Bohemia, Pilsen, Univerzitní 2732/8, 301 00 Pilsen, Czech Republic

**Keywords:** high-entropy alloy, aluminide, silicide

## Abstract

Novel high-entropy (multi-principal elements) alloy based on Fe-Al-Si-Ni-Ti in equimolar proportions has been developed. The alloy powder obtained by mechanical alloying is composed of orthorhombic FeTiSi phase with the admixture of B2 FeAl. During spark plasma sintering of this powder, the FeSi phase is formed and the amount of FeAl phase increases at the expense of the FeTiSi phase. The material is characterized by a high compressive strength (approx. 1500 MPa) at room temperature, being brittle. At 800 °C, the alloy is plastically deformable, having a yield strength of 459 MPa. The wear resistance of the material is very good, comparable to the tool steel. During the wear test, the spallation of the FeSi particles from the wear track was observed locally.

## 1. Introduction

Conventional alloys are based on one main element, to which various alloying elements are added according to confer desirable properties to materials. These traditional alloys include Fe alloys (mainly steels), Al alloy (such as durals or silumines), Mg or Ti alloys, and also Ni alloys. Of course, there are a number of such systems, but looking at the periodic table of elements, it is clear that this “series” is limited. New approaches are needed if the possibilities for discovering new materials (alloys) are to be significantly expanded. One possibility is to mix several main elements in high, often equimolar, concentrations. Background work began much earlier, but the first results about such alloys were not published until 2004 [1,2,3], and because this approach was in sharp contrast to traditional practice, it attracted considerable attention. Alloys composed of five or more principal elements in equimolar ratios were named “high-entropy alloys” (HEA) [1], however, we can also come across other names such as multi-component alloys, multi-principal-element alloys, or compositionally complex alloys as summarized in Miracle’s publication [4]. The basic definition of HEA is relatively loose and includes a large number of systems. HEAs do not need be equimolar, but the concentration of each major element must be between 3–35 at.%. The definition also places neither any bounds on the magnitude of entropy nor the requirement on the single-phase composition [1,4]. However, the first developers expected a single-phase, therefore, at present some authors and researchers are trying to separate alloys that meet only the requirement for concentration range and number of elements, but contain more than one phase, those alloys called compositionally complex alloys (CCA) [5]. However, inconsistencies and unclear definitions lead to overlapping and confusing names. The HEAs and refractory HEAs are characterized by very good mechanical properties, such as a very good combination of strength and ductility [6,7] and corrosion resistance [8,9]. However, the known HEAs contain the elements listed as critical raw materials, such as cobalt, titanium, tantalum, or niobium [10].

Powder metallurgy methods have become more and more popular not only in the case of conventional materials, such as steel and aluminum alloys, but also in the field of high-entropy alloys and intermetallics [11,12]. Among the methods, which enable achieving ultrafine-grained materials, mechanical alloying (MA) for the powder preparation and spark plasma sintering (SPS) are of high preference. The high versatility of the MA process and its non-equilibrium nature and ability to produce nanostructured powders are highly welcome by scientists in the last few decades. Mechanical alloying produces alloy powders from elemental ones by the means of high-energy milling, using ball mills, attritors, or other highly efficient milling devices, by the series of processes comprising deformation and cold welding of the powders, their severe plastic deformation, and deformation strengthening, crushing and also their heating by the kinetic energy of the collisions and the friction, supporting the mutual reactions of the components [13]. Spark plasma sintering is nowadays one of the most efficient sintering methods, which enables fast sintering, thus enabling the preservation of the nanostructured nature of the powders as much as possible. The rapid achievement of the sintering temperature and quick sintering process are achieved by the combination of uniaxial pressing and passage of the high pulsed direct current through the sample [14].

The iron aluminides (FeAl and Fe_3_Al) are considered or already applied in many high-temperature applications. Since the 1950s, when the Pyroferal, Tchugal, and Thermagal Fe-Al-C alloys have been developed [15], these materials underwent significant development. The concept of carbon-containing alloys was considered by many authors as problematic, because the Al_4_C_3_ carbide, which is one of the constituents of these materials, hydrolyzes in humid or acidic environments to methane [16], which leads to the destruction of the component. Therefore, the carbon-free alloys started to be developed, being alloyed by many alloying elements, such as chromium, niobium, or zirconium [17,18,19]. Boron was found to improve the fracture toughness of these alloys [20].

In our previous research, we focused on Fe-Al-Si-based alloys with various ratios between aluminum and silicon. These alloys reached a very high strength (up to 2000 MPa), when prepared by the combination of mechanical alloying and spark plasma sintering, maintaining a very fine structure. The alloys were also characterized by excellent oxidation resistance, much better than for binary Fe-Al alloys. However, their fracture toughness was very low, just up to 3.5 MPa m^1/2^ [21]. Surprisingly, the toughness was higher for FeAl20Si20 (high-silicon) alloy than for FeAl35Si5 (low-silicon) one, even though the FeAl35Si5 alloy is the material based on the FeAl matrix with a minor amount of the Fe_3_Si phase, while the FeAl20Si20 alloy contained brittle Fe-Al-Si phase and Fe_3_Si and FeSi silicides [21], both being considered as more brittle than FeAl [22,23]. Therefore, this work brings a new concept—a high entropy alloy based on aluminum, silicon, iron, and the element stabilizing aluminide phase (nickel) [24] and the silicide-forming one (titanium), which could solve the above-mentioned problems of the Fe-Al-Si based alloys.

## 2. Materials and Methods

The FeAlSiNiTi alloy was prepared from elemental, pure powders, which were mixed in appropriate amounts (see in Table 1) forming 5 g powder batches for mechanical alloying (MA). The following powders were used to prepare the blend for MA: Fe (purity 99.9%, particle size < 44 μm (Strem Chemicals, Newburyport, MA, USA), Al (purity 99.7%, particle size < 44 μm, Strem Chemicals), Si (purity 99.5%, particle size < 44 μm (Alfa Aesar, Haverhill, MA, USA), Ni (purity 99.99%, particle size < 150 μm, Strem Chemicals) and titanium (purity 99.5%, particle size < 100 μm, Strem Chemicals). The process of MA was carried out in a planetary ball mill—Retsch PM100 (Retsch, Haan, Germany), where the milling vessel/jar and also milling balls were made of/from AISI 420 stainless steel. After the powder mixture was placed in the vessel/jar, it was sealed and filled with a protective argon atmosphere. Argon was maintained to suppress the oxidation during the process. Other conditions were as follows: milling time of 4 or 8 h, every 30 min change of rotation direction, the rotational velocity of 400 rpm, and the ball-to-powder weight ratio of approx. 60:1. The milling was running continuously for the whole period of time, without any break.

Furthermore, only powder that was mechanically alloyed for 4 h was used. The material prepared by this way was compacted to 20 mm diameter cylindrical samples by spark plasma sintering (SPS, FCT Systeme, HP-D10, Rauenstein, Germany) at a temperature of 1000 °C with a pressure of 48 MPa for 10 min. However, during the process, the heating rate was controlled. Until 900 °C, a heating rate of 300 °C/min was used. After reaching 900 °C, the heating rate was reduced to 100 °C/min to avoid the excessive overheating of the sample during sintering. In order to reduce the thermal stress-strains and to avoid the cracking of the sample, the cooling rate was defined as 50 °C/min down to 300 °C. After that, the samples were cooled freely.

The phase composition of milled powders and also of the compacted sample was examined by X-ray diffraction (XRD) analysis (X’Pert Pro diffractometer, PANalytical, Almelo, The Netherlands) using CuK radiation with the wavelength of 0.154060 nm. The XRD patterns were acquired in the range from 5 to 90°. Microstructure of the material was observed using light microscopy (Olympus PME-3, Tokyo, Japan), and then investigated by VEGA 3 LMU (TESCAN, Brno, Czech Republic) scanning electron microscope (SEM) after etching by modified Kroll’s reagent (10 mL HNO_3_, 5 mL HF, 85 mL H_2_O). The electron microscope is equipped with an energy-dispersive spectrometer (EDS) X-max 20 mm^2^ (Oxford Instruments, High Wycombe, UK) enabling local analysis of elemental composition and acquisition of elemental distribution maps. Image analysis has been carried out by the means of ImageJ 1.53e software.

Vickers hardness with a load of 30 kg was measured on a standard hardness tester. The test was carried out with a dwell time of 10 s. The spacing between the indents was more than three times the length of the diagonals. The average (micro)hardness value was calculated from a minimum of six values obtained on one sample. The compressive tests were performed on cubic samples with the edge length of 2.5–3 mm using a universal testing device LabTech 5.250SP1-VM (Labortech s.r.o, Opava, Czech Republic) at laboratory temperature and 800 °C. The stain rate ranged from 0.160 to 0.163 mm/min. Since the alloys could be potentially applied as high-temperature materials, e.g., parts of hot presses or combustion engine valves, it is needed to know especially the properties at the room temperature (to prevent the damage during the montage and the cold operation) and at the service temperature. Therefore, we focused on these two conditions of the compressive tests. The direction of the applied pressure was the same as the direction of pressing during compaction. Tribological tests were done on the polished sample using a “pin-on-disc” setup. The tests were performed on a TRIBOtester instrument (Tribotechnic, Clichy, France) at laboratory temperature and standard humidity. The Al_2_O_3_ ball with a diameter of 6 mm was moving along the sample in the linear reciprocating mode with a speed of 10 mm∙s^−1^. The normal force acting on the ball was 5 N, and the total distance was 20 m. Since the material is relatively hard, we selected an aluminum oxide ball among the standardized materials used for this kind of test. These conditions were used in our previous works dealing with aluminide and silicide-based materials [21,25]. The profile and depth of the wear track were then measured using a DH-15 profilometer (Diavite, Bülach, Switzerland) with the skidless probe (Tribotechnic, Clichy, France) at three locations (approximately at ¼, ½ and ¾ of the track length).

## 3. Results

### 3.1. Phase Composition and Microstructure

**Figure 1:**
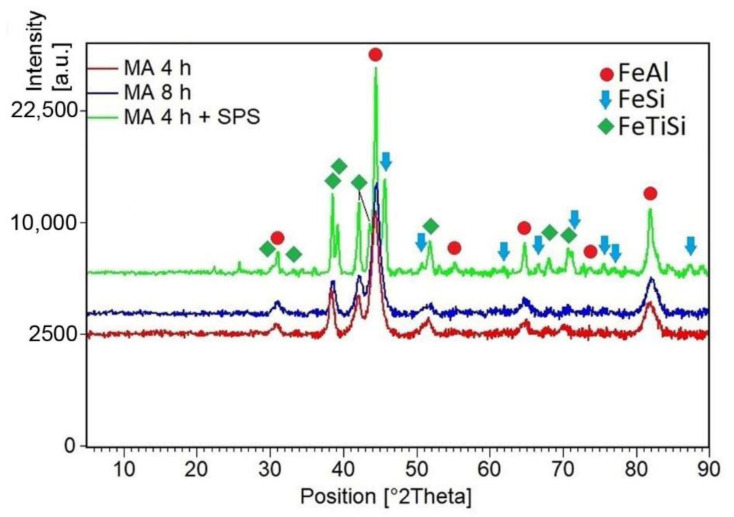
Phase composition vs. the preparation conditions.

During the optimization of the mechanical alloying process, two milling times were compared, 4 vs. 8 h. As can be seen in Figure 1 both times lead to the same results in terms of phase composition, see Figure 1. Both milled powders are composed of a major amount of a phase listed as FeTiSi phase with orthorhombic structure (Ima2, a = 6.9970 Å, b = 10.8300 Å, c = 6.2870 Å) with the minor admixture of a FeAl phase (cubic, Pm-3m, a = 2.9030 Å). Since the longer milling duration did not cause a significant change in the phase composition, the powder milled for 4 h was subjected to sintering. During sintering, the phase composition changed slightly, see Figure 1. Both FeTiSi and FeAl phases are conserved in the sample, but the FeSi phase (cubic, P213, a = 4.4373 Å) formed during thermal exposure in the SPS process. The crystallite size, estimated from the XRD patterns by the means of the Sherrer’s method, increases with the milling duration from 7.5 nm for 4 h milling to 10.9 nm for 8 h of milling, probably due to the dissipated heat from the milling process, which can cause a significant increase of the temperature in the milling vial. Spark plasma sintering, being carried out at 1000°C, caused a stronger increase of this parameter to 27.7 nm. However, it has to be noted that the results of Sherrer’s method are generally affected by the lattice stain, which can be highly expected in the material after milling.

**Figure 2:**
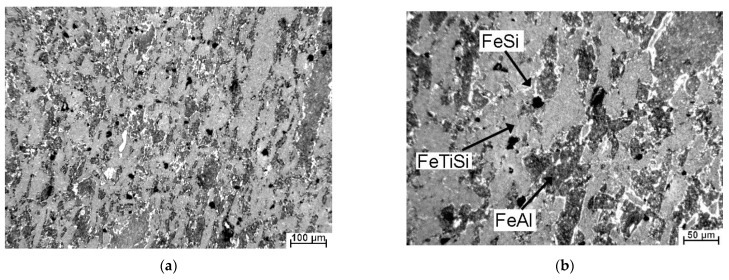
Microstructure of bulk FeAlSiNiTi produced by MA + SPS (**a**), higher magnification detail (**b**). The phases are labeled based on the EDS results below.

The microstructure of the sintered alloy is shown by the optical micrograph in Figure 2. Even though an ultrafine-grained structure is usually expected when the sample is prepared by the combination of MA and SPS, this material is characterized by a relatively coarse structure. The dark-gray areas probably belong to the aluminide (FeAl) phase, the medium-gray matrix is the phase identified as FeTiSi, while the lighter fine particles are highly probably the silicides (identified as FeSi by XRD), which were formed during the sintering process. The black spots are the pores, which result from incomplete sintering probably due to random geometries of mechanically alloyed powder particles and poor compressibility of the alloy powder. The area fraction of FeAl phase on the optical micrograph (approximately corresponds to the volume fraction) is approx. 35%, while FeSi occupies just 4% and the porosity reaches approx. 1%, as determined by the image analysis.

**Figure 3:**
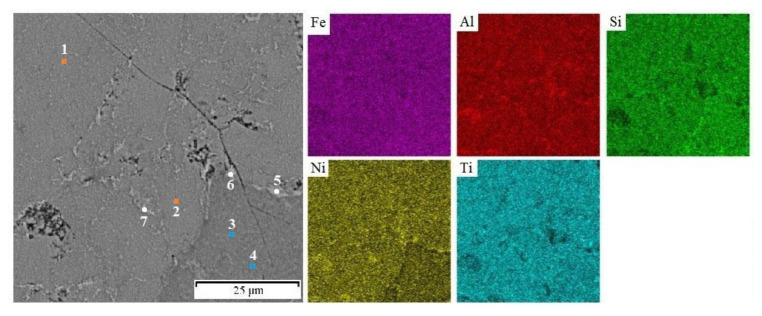
Element distribution maps of the sample. The numbers in the left image indicate the points of the EDS analyses presented in Table 2.

Local chemical analysis carried out by the EDS method (Table 2) enabled the determination of the chemical composition of the individual phases. The matrix phase identified by XRD as FeTiSi in fact contains all of the elements in an approximately equiatomic ratio (Figure 3 and Table 2, points 1 and 2). The phase identified as FeAl is strongly enriched by nickel (points 5–7 in Table 2), which is a strong aluminide-forming element. The amount of titanium in this phase is much lower because titanium rather occupies the silicide phase (points 3 and 4) in Figure 3 and Table 2. In the FeSi phase, a relatively high amount of aluminum has been determined. It could be caused by the fact that the silicide particles are rather small and hence the chemical analysis results are influenced by the surrounding matrix due to the interaction volume in SEM andEDS.

### 3.2. Mechanical and Tribological Properties

**Figure 4:**
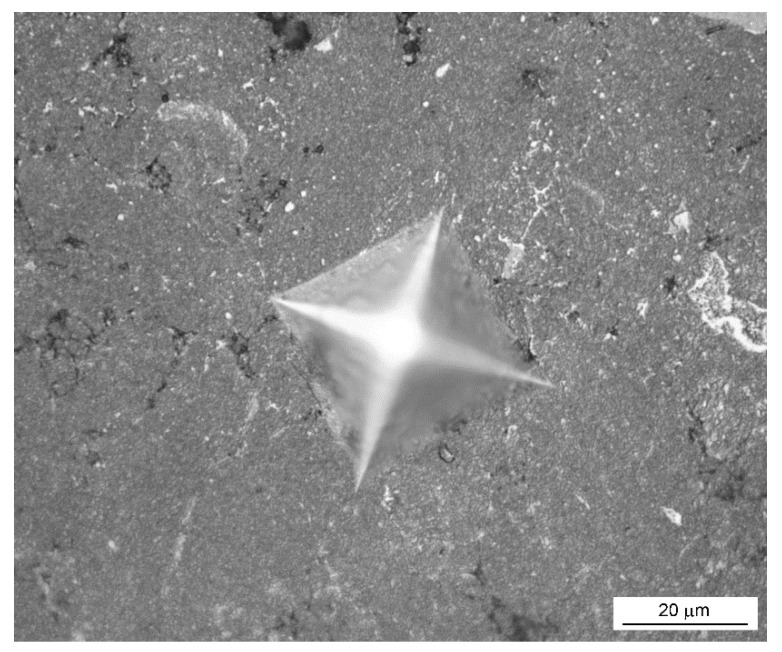
Appearance of the indentation by Vickers method (load of 1 kg).

**Figure 5:**
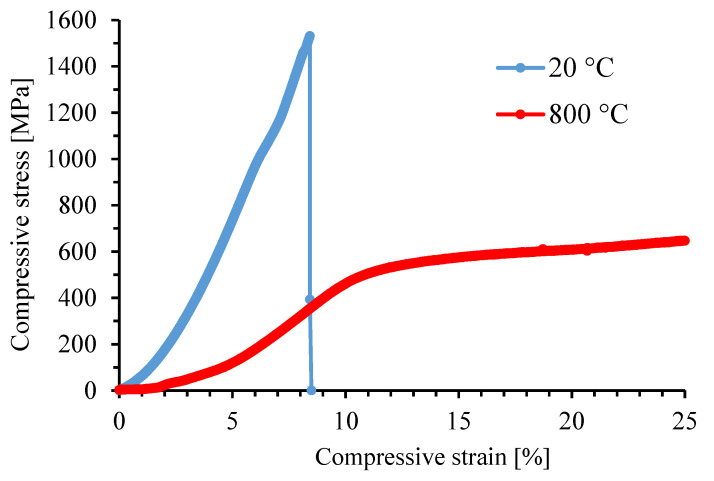
Stress-strain curves in compression. Tested at 20 and 800 °C.

The room-temperature hardness of the alloy reaches approx. 630 HV 30 (Table 3), which exceeds the hardness values of common alloys used for high-temperature service. The fracture toughness could not be measured by the Palmquist method due to the absence of the cracks around the indentation (Figure 4). It implies that the toughness is surely higher than that of the previously studied ternary FeAl20Si20 and FeAl35Si5 (in wt. %) alloys and the quaternary alloys derived from them. Compared to other used materials (Inconel 601, β-titanium alloys, stainless steels), the FeAlSiNiTi alloy also offers excellent ultimate compressive strength (UCS) of 1528 MPa at room temperature. However, the disadvantage is that the material behaves as brittle at room temperature, i.e., is not capable of plastic deformation (Figure 5).

The compression tests at elevated temperatures, specifically at 800 °C, brought an interesting change in the behavior of the material. The previously brittle alloy changed its behavior and became plastic. This behavior is typical for many systems based on intermetallics, where the different slip systems can be activated at high temperatures [26]. Since the sample did not break during the test and only the regions of elastic and plastic deformation were observed, it was not possible to evaluate the UCS value. The yield strength of this material at 800 °C reaches the value of 459 MPa.

**Figure 6:**
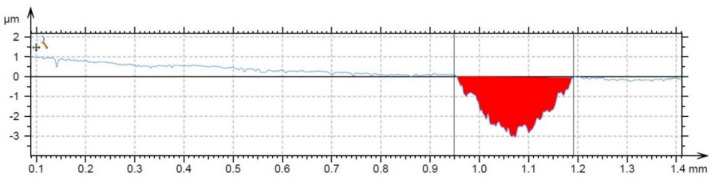
The profile of the wear track after the wear resistance test against the alumina ball.

**Figure 7:**
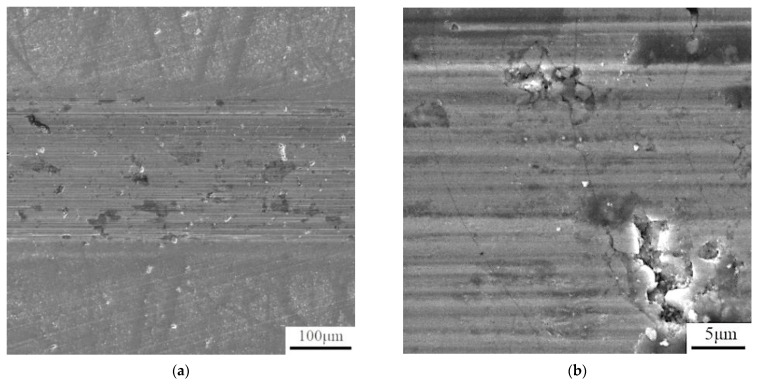
Analysis of the wear track by SEM (**a**), higher-magnification detail (100 μm) (**b**), higher-magnification detail (5 μm).

The wear resistance tests, carried out on the polished surfaces (see Table 4 for surface roughness Ra value) against the alumina ball, showed that the alloy is highly wear-resistant. The value of a friction coefficient is lower than for the dry sliding wear of a steel or nickel alloy under the same conditions [27]. The profile of the wear track shows that the abrasion of the material took place, leaving the abraded track. No signs of plastic deformation are visible in the vicinity of the wear track (Figure 6). From the SEM observation of the wear tracks (Figure 7), it can be seen that the particles were scaled off from the material, which subsequently supported its abrasive wear. Due to the morphology of the traces left after the particles, it could be expected that the released brittle phase is FeSi based silicide.

## 4. Discussion

The initial idea of the work was to combine silicon and aluminum with the mixture of elements, which form both silicides and aluminides. Among the used alloying elements, nickel was previously found by both thermodynamic calculations and experiments to form rather aluminides than silicides [24], acting as aluminide stabilizers. On the other hand, titanium is a silicide-former [28]. Iron was found in our previous works to be “neutral”, i.e., to form aluminides, silicides, or even Fe-Al-Si phases, depending on the Fe:Al:Si ratio [29]. When the ternary Fe-Al-Si alloys and their quaternary alternatives with the addition of Ni or Ti were prepared by mechanical alloying, a high degree of a mutual substitution by aluminum and silicon in aluminides and silicides was observed, respectively [21]. So we expected based on previous results that when the above-mentioned elements would be combined in equimolar proportions, we will obtain a highly substituted aluminide, silicide, or Fe-Al-Si based phase, where iron would be partially substituted by nickel and titanium. However, the latter possibility was considered as the less probable one, because both nickel and titanium decreased the amount of the ternary Fe-Al-Si phases in the mechanically alloyed materials [25]. In reality, the experiment led to the formation of a major amount of a phase, which was identified as the orthorhombic FeTiSi phase, being constituted by all used elements in approximately equimolar ratios. So, we can expect that the material could be a single-phase one, composed by this phase in an ideal case. In reality, we obtained also a minor amount of FeAl phase (ordered phase with B2 structure). Quite surprisingly, material based on the orthorhombic FeTiSi phase reaches higher toughness than the previously studied Fe-Al-Si alloys after sintering [21], even though the Fe-Al and Fe-Si phases in Fe-Al-Si alloys are the cubic ones, which are usually connected with higher plasticity due to high symmetry and corresponding higher number of available slip systems [30,31]. The higher toughness was assessed based on the indentation tests, where there were no cracks visible around the indentation in the case of this new alloy.

The mechanical properties are generally very promising, combining high ultimate compressive strength at the room temperature of approx. 1500 MPa and the yield strength of 459 MPa at 800 °C. This value is much higher than in the case of the Inconel series alloys, as well as the previously tested Fe-Al-Si alloys [21]. The wear rate is reaching a very good value, even though the release of the hard FeSi particles was observed on the wear track. This phenomenon is probably caused by the brittle nature of iron silicide. This phase formed during the sintering process, so further optimization of the sintering conditions, which would potentially avoid the formation of this phase, will probably also increase the wear resistance, as well as the mechanical properties. Based on the determined properties, it can be expected that the new HEA will be applicable as a material for the manufacture of high-temperature tools, such as the parts of the hot presses or furnace elements.

## 5. Conclusions

The Fe-Al-Si-Ni-Ti high-entropy (multi-principal elements) alloy was successfully prepared by the combination of mechanical alloying and spark plasma sintering. While the mechanically alloyed powder was composed mostly of the orthorhombic FeTiSi phase with a minor amount of a B2 FeAl phase, the sintering caused the increase of the content of FeAl phase, as well as the formation of fine particles of FeSi. The matrix FeTiSi phase contained all of the alloying elements, while the FeAl phase was strongly enriched by nickel. Titanium was bound in the FeSi silicide phase. The alloy is considerably brittle at room temperature but reaches the ultimate compressive strength higher than 1500 MPa and very good wear resistance. At 800 °C, the alloy behaves plastically having the compressive yield strength of 459 MPa. Due to this combination of properties, the alloy could be potentially used for the manufacture of high-temperature tools, such as the parts of the hot presses.

## Figures and Tables

**Table 1:** Content of element in FeAlSiNiTi alloy.

Content	Element				
	Fe	Al	Si	Ni	Ti
at. [%]	20.0	20.0	20.0	20.0	20.0
wt. [%]	25.7	12.4	12.9	27.0	22.0

**Table 2:** Results of the SEM + EDS analysis of the points marked in Figure 3.

Element Points	Fe	Al	Si	Ni	Ti	**Corresponding Phase**
	at. [%]					
1	20.4	22.4	18	20.3	18.9	FeTiSi
2	21.4	21.1	19	19.2	19.3	FeTiSi
3	24.2	19.9	20.8	15.7	19.4	FeSi
4	23.2	21.1	20.7	15.5	19.5	FeSi
5	21.2	29.8	11.6	26.0	11.4	FeAl
6	17.8	32.8	12.7	27.0	9.7	FeAl
7	17.6	31.6	13.9	25.4	11.5	FeAl

**Table 3:** Mechanical properties of alloy.

**FeAlSiNiTi**	**HV30** **[-]**	**UCS** **[MPa]**
	629 ± 82	1528

**Table 4:** Results of the tribological testing done at laboratory temperature (Ra—surface roughness, w—wear rate, f—friction coefficient).

**FeAlSiNiTi**	**Ra** **[μm]**	**w** **[mm^3^N^−1^m^−1^]**	**f** **[-]**
	0.0375	24.44 × 10^−6^	0.674

## Data Availability

Data are stored by the authors, not available publicly.
